# Cav1/EREG/YAP Axis in the Treatment Resistance of Cav1-Expressing Head and Neck Squamous Cell Carcinoma

**DOI:** 10.3390/cancers13123038

**Published:** 2021-06-18

**Authors:** Mickaël Burgy, Aude Jehl, Ombline Conrad, Sophie Foppolo, Véronique Bruban, Nelly Etienne-Selloum, Alain C. Jung, Murielle Masson, Christine Macabre, Sonia Ledrappier, Hélène Burckel, Carole Mura, Georges Noël, Christian Borel, François Fasquelle, Mihaela-Alina Onea, Marie-Pierre Chenard, Alicia Thiéry, Monique Dontenwill, Sophie Martin

**Affiliations:** 1 Laboratory of Bioimaging and Pathology, University of Strasbourg, UMR7021 CNRS, 67401 Illkirch, France; m.burgy@icans.eu (M.B.); aude.jehl@etu.unistra.fr (A.J.); ombline.conrad@etu.unistra.fr (O.C.); sophie.foppolo@unistra.fr (S.F.); veronique.bruban@unistra.fr (V.B.); nelly.etienne-selloum@unistra.fr (N.E.-S.); monique.dontenwill@unistra.fr (M.D.); 2 Department of Medical Oncology, Institut de Cancérologie Strasbourg Europe, 67200 Strasbourg, France; C.borel@icans.eu; 3 Department of Pharmacy, Institut de Cancérologie Strasbourg Europe, 67200 Strasbourg, France; 4 Laboratory STREINTH (Stress Response and Innovative Therapies), Inserm IRFAC U1113, Université de Strasbourg, 67200 Strasbourg, France; a.jung@icans.eu (A.C.J.); CMacabre@strasbourg.unicancer.fr (C.M.); s.ledrappier@icans.eu (S.L.); 5 Laboratory of Tumor Biology, Institut de Cancérologie Strasbourg Europe, 67200 Strasbourg, France; 6 UMR7242 Biotechnologie et Signalisation Cellulaire, Ecole Supérieure de Biotechnologie de Strasbourg, 67412 Illkirch, France; murielle.masson@unistra.fr; 7 Paul Strauss Comprehensive Cancer Center, Radiobiology Laboratory, Institut de Cancérologie Strasbourg Europe (ICANS), Strasbourg University, UNICANCER, 67000 Strasbourg, France; h.burckel@icans.eu (H.B.); C.mura@icans.eu (C.M.); g.noel@icans.eu (G.N.); 8 Paul Strauss Comprehensive Cancer Center, Institut de Cancérologie Strasbourg Europe (ICANS), Department of Radiation Oncology, Unicancer, 67200 Strasbourg, France; 9 Institut Pathology, University Hospital of Lausanne, 1011 Lausanne, Switzerland; Francois.Fasquelle@chuv.ch; 10 Department of Pathology, Strasbourg University Hospital, 67200 Strasbourg, France; Mihaela.ONEA@chru-strasbourg.fr (M.-A.O.); Marie-Pierrette.CHENARD@chru-strasbourg.fr (M.-P.C.); 11 Department of Public Health, Institut de Cancérologie Strasbourg Europe, 67200 Strasbourg, France; athiery@strasbourg.unicancer.fr

**Keywords:** head and neck cancer, biomarkers, EGFR therapy

## Abstract

**Simple Summary:**

The EGFR-targeting antibody cetuximab (CTX) combined with radiotherapy has been proven effective for the treatment of locally advanced head and neck squamous cell carcinoma (LA-HNSCC). Due to resistance to CTX, some patients do not benefit from the treatment and recurrence is observed. As caveolin-1 (Cav1) has been reported to affect the EGFR pathway, we aimed to elucidate how it might affect the response to CTX-radiotherapy. We showed that Cav1 expression conferred surviving, growing and motile capacities that protect cells against the combination of CTX-radiotherapy. The protecting effects of Cav1 are mediated by the Cav1/EREG/YAP axis. We also showed in a retrospective study that a high expression of Cav1 was predictive of locoregional relapse of LA-HNSCC. Cav1 should be taken into consideration in the future as a prognosis marker to identify the subgroup of advanced HNSCC at higher risk of recurrence, but also to help clinicians to choose the more appropriate therapeutic strategies.

**Abstract:**

The EGFR-targeting antibody cetuximab (CTX) combined with radiotherapy is the only targeted therapy that has been proven effective for the treatment of locally advanced head and neck squamous cell carcinoma (LA-HNSCC). Recurrence arises in 50% of patients with HNSCC in the years following treatment. In clinicopathological practice, it is difficult to assign patients to classes of risk because no reliable biomarkers are available to predict the outcome of HPV-unrelated HNSCC. In the present study, we investigated the role of Caveolin-1 (Cav1) in the sensitivity of HNSCC cell lines to CTX-radiotherapy that might predict HNSCC relapse. Ctrl- and Cav-1-overexpressing HNSCC cell lines were exposed to solvent, CTX, or irradiation, or exposed to CTX before irradiation. Growth, clonogenicity, cell cycle progression, apoptosis, metabolism and signaling pathways were analyzed. Cav1 expression was analyzed in 173 tumor samples and correlated to locoregional recurrence and overall survival. We showed that Cav1-overexpressing cells demonstrate better survival capacities and remain proliferative and motile when exposed to CTX-radiotherapy. Resistance is mediated by the Cav1/EREG/YAP axis. Patients whose tumors overexpressed Cav1 experienced regional recurrence a few years after adjuvant radiotherapy ± chemotherapy. Together, our observations suggest that a high expression of Cav1 might be predictive of locoregional relapse of LA-HNSCC.

## 1. Introduction

Head and neck squamous cell carcinoma (HNSCC) represents the fifth most common cancer worldwide, with an annual incidence and mortality estimated to be approximately 600,000 and 375,000 cases, respectively [1]. Locally advanced HNSCC (LA-HNSCC, stage III/IV) represents about 60% of patients at diagnosis. They require primary surgery followed by adjuvant (chemo)radiotherapy or definitive chemoradiotherapy. Despite such therapeutic strategies, the 3-year survival rate does not exceed 60% due to regional recurrence or distant metastasis that occurs in 50% of patients. In clinicopathological practice, it is difficult to assign patients to classes of risk because no reliable biomarkers are available to predict the outcome of HPV-unrelated HNSCC.

Cetuximab (CTX) is a human/murine chimeric IgG1 monoclonal antibody that binds to the epidermal growth factor receptor (EGFR). CTX promotes EGFR internalization, preventing the downstream signal [2,3]. Bonner’s trial [4,5] showed a significant improvement in locoregional control (three-year rates of locoregional control: 47% with radiotherapy plus cetuximab vs. 34% with radiotherapy alone) and median overall survival (49 vs. 29 months) for patients treated with CTX and radiotherapy vs. radiotherapy alone. These results led to the FDA approval of CTX in combination with radiotherapy for the treatment of LA-HNSCC [6,7]. The GORTEC 2007-01 phase III randomized trial showed no survival benefit associated with CTX combined with chemoradiation despite significant gains in locoregional control. This combination is therefore not recommended as treatment for HNSCC [8]. To date, CTX combined with radiotherapy is the only targeted therapy that has been proven effective for the treatment of LA-HNSCC patients ineligible for cisplatin. Vermorken and colleagues [9] showed the benefits of combining CTX with cisplatin/carboplatin and 5-FU (EXTREME regimen) vs. chemotherapy alone for recurrent or metastatic HNSCC (R/M-HNSCC). In 2019, the KEYNOTE-048 study demonstrated the superiority of pembrolizumab alone or in combination with platinum and 5-FU in all patients vs. EXTREME [10]. This result set immunotherapy as the new standard of care (SOC) in the R/M setting. However, the survival benefit of immunotherapy ± chemotherapy is not clearly demonstrated for all subgroups and overall survival seems to be less important for the PD-L1 combined positive score (CPS) <1. Thus, indication of pembrolizumab combination is restricted by the European Medicines Agency (EMA) to patients whose disease expresses PD-L1 with CPS ≥1, with an ongoing role for CTX in the first line for the CPS <1 population. Unfortunately, some patients do not benefit from CTX treatment, and others show recurrences soon after the end of the treatment. Both cases suggested an intrinsic or therapeutically acquired resistance to CTX. Extensive studies have sought to understand the mechanisms involved.

Caveolin-1 (Cav1) is one of the main constituents of lipid domains known as caveolae. Cav1 triggers, and plays a key role in, all the features described as the hallmarks of cancer. Whether Cav1 is an oncogene or a tumor suppressor is debated; however, the answer may be related not only to the levels expressed but also to its function, localization (tumor or stromal cells), type and stage of cancer, or physical forces within the environment of the tumor [11,12]. In HNSCC, low or no expression of Cav1 was reported to be predictive of metastasis-prone HNSCC [13,14,15]. We showed that the disappearance of Cav1 triggered epithelial to mesenchymal transition associated with the expression of integrins and MMPs responsible for the motile and invasive processes promoting metastasis [13]. Cav1 was initially described as a negative regulator of EGFR [16]. However, Cav1 can also promote EGFR activation and facilitate downstream signal transduction by relocating EGFR close to its signaling partners. Cav1 also modifies the half-life and cell surface availability of EGFR [17,18,19,20,21,22,23,24]. It is therefore not surprising that Cav1 might be predictive of anti-EGFR drug efficacy or failure [22,25,26,27,28]. How Cav1 overexpression affects the response to treatment and tumor evolution is only sparsely documented in HNSCC. We have highlighted here that the overexpression of Cav1 confers survival capacities on cells that remain proliferative and motile when exposed to CTX-radiotherapy. Such resistance might explain locoregional recurrence in Cav1-overexpressing tumors.

## 2. Materials and Methods

### 2.1. Cell Culture, Transfection and Drugs

SCC9 and CAL33 cell lines were purchased from ATCC (LGC Standards S.a.r.l., F-67123 Molsheim Cedex France) and DSMZ (Leibniz Institute DSMZ-German Collection of Microorganisms and Cell Cultures GmbH, 38124 Braunschweig, Germany (authenticated by STR profiling). All cell lines tested negative for mycoplasma contamination. SCC9 cells were grown in DMEM-F12 (PAN Biotech, Aidenbach, Germany) supplemented with 2.5 mM ultraglutamine, 15 mM HEPES, 400 ng/mL hydrocortisone (Sigma, Lyon, France) and 10% FBS (Gibco, DUTSCHER SAS, BRUMATH Cedex, France). CAL33 cells were grown in DMEM (PAN Biotech) supplemented with 2 mM ultraglutamine, 0.5 mM sodium pyruvate and 10% heat-inactivated FBS (Gibco). To overexpress Cav1, SCC9 and CAL33 cell lines were electroporated (Neon^®^ electroporation transfection system, Invitrogen, Thermo Fischer Scientific, Illkirch, France) with pEZ-M68_Ctrl_ or pEZ-M68_Cav1_ expression vectors (GeneCopoeia^TM^, Tebu-Bio, Le Perray-en-Yvelines Cedex, France; CAL33C_trl_ and CAL33_Cav1_) following the manufacturer’s instructions. Stable cell lines were obtained after selection with puromycin. The overexpression of Cav1 was confirmed by Western blot. EREG expression was downregulated by transfecting CAL33 cells with 50 nM siRNA_EREG_ (and the respective control siRNA_Ctrl_, SMARTPool Dharmacon, Fischer Scientific, Illkirch Cedex, France; CAL33_siRNA-Ctrl_ and CAL33_siRNA-Cav1_) using Lipofectamine 2000^TM^ (Invitrogen, Thermo Fischer Scientific, France). Efficient EREG silencing was determined by RT-qPCR. To overexpress YAP, CAL33 cells were transfected with 2 µg of Flag-YAP expression vectors (and the respective control mock, a generous gift from Dr Masson, ESBS, France; CAL33_Mock_ and CAL33_Flag-YAP_) using Lipofectamine 2000^TM^ (Invitrogen). The overexpression of YAP was confirmed by Western blot. When indicated, cells were treated with 10, 30 and 50 nM of CTX (Erbitux^™^, 5 mg/mL, Merck, ICANS, France), a monoclonal antibody classified as an antineoplastic agent. The concentrations were consistent with those achieved in patients (Merck information product sheet).

### 2.2. Irradiation

Experiments were performed in “replating conditions” (cells were treated before being plated for further experiments) that might better reflect the situation of normal fractionation [29]. Irradiation was delivered at room temperature using single doses of γ-rays (Cesium^137^) with a Biobeam GM 8000 irradiator (GSM GmbH, Leipzig, Germany) at the Paul Strauss Cancer Center (ICANS, Strasbourg, France) at a dose-rate of 2.5 Gy/minute. The applied doses ranged from 0 to 10 Gy. Cells were kept at 37 °C in 5% CO_2_ for 24 h before being seeded for further experiments. When indicated, cells were treated with CTX (10, 30 or 50 nM) 2 h prior to irradiation.

### 2.3. Clonogenic Survival Assay

Twenty-four hours after treatments (10, 30 or 50 nM cetuximab alone, 0–10 Gy irradiation alone or two hours cetuximab pretreatment before irradiation), cells were seeded (250, 500 and 1000 cells/2 mL) in 6-well plates and allowed to grow for 10 days. Cells were stained with crystal violet at 0.1% (Sigma-Aldrich, St Quentin Fallavier Cedex, France). Colonies were counted to determine the plating efficiency (PE) and the surviving fraction (SF). PE = number of surviving cells/number of cells plated. SF = PE of the experimental group/PE of the control group.

### 2.4. IncuCyte^®^ Assay

After CTX and irradiation alone or in combination, cells were seeded (1000–2000 cells/200 µL/well) in 96-well plates. Plates were kept at 37 °C for 7 days. Growth (monitored by analyzing the area occupied by cells (% confluence)), cell health and morphology were monitored for 7 days. The percentage of confluence was determined using the IncuCyte^®^ analysis software after normalization to day 0 (Essen BioScience, Sartorius, Goettingen, Germany).

### 2.5. Western Blot

After treatments, cells were lysed with lysis buffer (1% Triton, 100 nM NaF, 10 mM Na_4_O_7_P_2_, 1 mM Na_3_VO_4_, protease inhibitor cocktail (Roche, Sigma-Aldrich, St Quentin Fallavier Cedex, France) in PBS) for 20 min at 4 °C and then sonicated. The supernatant was recovered by centrifugation at 14000 rpm for 10 min at 4 °C. A total of 1 to 20 μg of protein was separated on a 4–20% TGX-denaturing polyacrylamide gel (SDS-PAGE Bio-Rad, Marnes-La-Coquqette, France) and transferred to a polyvinylidene difluoride (PVDF) membrane (Amersham, Sigma-Aldrich, St Quentin Fallavier Cedex, France). Blots were probed with various antibodies (see Table S1). Proteins were visualized with enhanced chemiluminescence using the LAS4000 imager and densitometry analysis was performed using ImageQuant Software (GE Healthcare, Tremblay-en-France, France).

### 2.6. Cell Cycle and Apoptosis Analysis

After treatment, cells were collected and centrifuged at 1500 rpm for 5 min, fixed in cold 70% ethanol and placed at −20 °C for at least 24 h. Cell cycle distribution and apoptosis were determined using propidium iodide/RNaseA staining (dilution 1/100, Merck Millipore, Molsheim, France). Cells were incubated for 30 min at room temperature in the dark before the fluorescence was analyzed using a flow cytometer (BD Accuri^®^ C6 Becton Dickinson, Rungis, France).

### 2.7. Metabolic Assay

After treatment, 20,000 cells were plated in a Seahorse XF Cell Culture microplate in XF growth medium (nonbuffered DMEM containing 10 mM glucose, 4 mM L-glutamine, and 2 mM sodium pyruvate). OCR (oxygen consumption rate) and ECAR (extracellular acidification rate) were measured using the mitochondrial stress test procedure under basal conditions and in response to 3.5 μM oligomycin, 0.5 and 1 μM fluoro-carbonyl cyanide phenylhydrazone (FCCP) and 14 μM rotenone + 14 μM antimycin A with the XFp Extracellular Flux Analyzer (Seahorse Bioscience, Agilent, Les Ulis, France). The metabolic profiles were analyzed using Seahorse software (Agilent, Les Ulis, France).

### 2.8. Sphere Evasion Assay

After treatments, 500,000 cells were resuspended in 1 mL of regular culture medium supplemented with 20% methylcellulose. Spheroids were formed using the hanging drop culture method. Drops of 20 µL cell suspension were placed onto the lids of 60 mm dishes which were inverted over the dishes. Dishes were cultured in humidified chambers (containing PBS) for 24 h to allow the formation of round aggregates. Spheroids were harvested and seeded in plastic 24-well plates (4 spheres/well) for an additional 24 h to allow evasion of cells from attached spheres. Pictures were taken using an Evos XI Core microscope (AMG, Thermo Fischer Scientific, Illkirch, France) at 10× magnification. The results were expressed, in pixels, as the evasion area of the cells relative to the area of the attached sphere (total area—area of the sphere) determined using ImageJ (https://imagej.nih.gov, access on 3 May 2021).

### 2.9. Human Tissue Samples

All tumor specimens (*n* = 173) were collected during the initial surgery and stored until use in the tumor Bank (Paul Strauss Cancer Center, Strasbourg, France). Informed consent was obtained from all subjects involved in the study. The collection of HNSCC samples was declared to the Bioethical unit of the French Ministry of Higher Education, Research and Innovation (Declaration DC-2013-1798), and was authorized by the same authority (AC-2018-3177, 22 November 2018). The management of patient data was declared and authorized by the French National Commission for Data Protection and Liberties (CNIL; 519013 and 601451). Patients from the northeastern region of France underwent initial surgical resection of their localized HNSCC between 2003 and 2013 at Saint Barbe Clinic (Strasbourg, France), followed by postoperative radiotherapy or chemoradiotherapy (cisplatin) at the Paul Strauss Cancer Center (Strasbourg, France) or the Civil Hospitals of Colmar or Mulhouse. Hematoxylin-eosin slides of paraffin-embedded tumor (FFPE) specimens were examined by two pathologists. All tumors were confirmed as squamous cell carcinomas. The inclusion criteria were: tumor localization (hypopharynx, oropharynx or oral cavity, HPV-negative), ≥T3 and/or ≥N2a with no clinical or radiographic evidence of distant metastases. The primary endpoints were metastatic disease and locoregional recurrence-free survival 3 years after surgery. Secondary endpoints included overall survival (OS), defined as the time from the surgery to the date of death or last follow-up. The recorded variables included age, Eastern Cooperative Oncology Group (ECOG) and Karnofsky Performance Score (KPS), comorbidities (Charlson comorbidity index), tumor stage, chemotherapy regimen in combination with radiotherapy, smoking and alcohol consumption, and follow-up data (survival data, biological parameters, and nutritional characteristics). For detailed patient demographics see Supplementary Data, Table S2.

### 2.10. Immunohistochemistry on Human Tissue Samples

The expression of Cav1 was evaluated by immunohistochemical (IHC) analysis using a Ventana Autostainer Automat (Ventana Medical Systems, Roche Tissue Diagnostics, Boulogne-Billancourt, France). Slides were prepared from formalin-fixed paraffin-embedded tumor specimens. Slides were stained for Cav1 (N-20 sc-894; Santa Cruz Biotechnology, Heidelberg, Germany; dilution 1/50) according to the manufacturers’ instructions. Signals were revealed with the ultraView Universal DAB Detection Kit (Ventana Medical Systems, Roche Tissue Diagnostics, Boulogne-Billancourt, France), according to the manufacturer’s instructions. All images were acquired with an Olympus BX60 with 20× or 40× objectives. Contrasts were uniformly adjusted on all images with Photoshop (Adobe) software (https://www.adobe.com; access on 3 May 2021). We used two different semiquantitative analyses of the IHC staining of Cav1. In the first category, tumors were classified into 4 categories according to the percentage of Cav1-positive carcinoma cells: 0 (0%), + (1–25%), ++ (26–75%) and +++ (>75%). In the second one, the histoscore (H-Score) was calculated as a percentage of different positive cells for Cav1 (0, 1, 2 or 3) using the formula (1 × (% cells 1) + 2 × (% cells 2) + 3 × (% cells 3)).

### 2.11. Immunohistochemistry on Cells

After treatment, cells were seeded in the Nunc Lab-Tek II CC^2^ 8-well Chamber Slide System at a density of 2000 per well and cultured for 2 days. Cells were then fixed in ice-cold methanol for 10 min and washed in PBS. After a 60 min blocking step in PBS/3% BSA/0.3% Triton X-100, cells were incubated overnight at 4 °C with EGFR antibody (#4267; Cell Signaling Technology, Ozyme, Saint-Cyr-L’Ecole, France; dilution 1/50). After washing in PBS, cells were incubated with appropriate secondary antibodies (Life Technologies; dilution 1/500) and DAPI (#D9542; Sigma-Aldrich, St Quentin Fallavier Cedex, France; 1 µg/mL). After washing in PBS, the slides were mounted using Fluoromount-G medium (#00-4958-02; Thermo Fisher Scientific, Illkirch, France). Images were acquired using a LEICA TCS SPE II confocal microscope (Leica Microsystems SA, Nanterre Cedex, France), with a 60 × magnification oil-immersion objective, and analyzed with ImageJ software (https://imagej.nih.gov, access on 3 May 2021).

### 2.12. Real-Time Quantitative PCR on Human Tissues Samples

Total RNA was extracted from frozen tumor tissues using DNA/RNA allprep minikits (Qiagen, Courtaboeuf Cedex, France), according to the manufacturer’s instructions. The integrity of the extracted RNA was verified on an Agilent 2100 Bioanalyzer (Agilent Technologies, Palo Alto, CA). RNA concentrations were measured using an ND-1000 NanoDrop spectrophotometer (Labtech, Palaiseau, France). Then, 0.5 µg of extracted RNA was used for cDNA synthesis using the Goscript reverse transcription system (Promega, Charbonnières-les-bains, France) according to the manufacturer’s instructions. One microliter of diluted cDNA, corresponding to either 5 or 1.25 ng of reverse transcribed RNA, was analyzed with SYBR Green (Roche, Meylan, France), in duplicate, using the LightCycler 480 real-time PCR system (Roche, Meylan, France). qRT-PCR data were analyzed using LightCycler^®^ 480 software (Roche, Meylan, France). Ct levels were normalized to the geometric mean of the Ct values of 2 internal controls (housekeeping genes): UBB (ubiquitin B) and RPLP0 (ribosomal protein large P0). The following primer pairs were used: CAV1 (5′-ACCGCGACCCTAAACACCTC-3′ and 5′-CCTTCCAAATGCCGTCAAAA-3′), RPLP0 (5′-GAAGGCTGTGGTGCTGATGG-3′ and 5′-CCGGATATGAGGCAGCAGTT-3′) and UBB (5′-GCTTTGTTGGGTGAGCTTGT-3′ and 5′-CGAAGATCTGCATTTTGACCT-3′).

### 2.13. Real-Time Quantitative PCR on Cells

RNA was extracted as previously described. mRNA expression was evaluated by relative quantitative RT-qPCR analysis using the StepOne Plus (Applied Biosystems, Fischer Scientific, Illkirch Cedex, France) FastSYBRGreen PCR detector. The primer pairs (Invitrogen, Fischer Scientific, Illkirch Cedex, France) were: CAV1 (5′-ACCGCGACCCTAAACACCTC-3′ and 5′-CCTTCCAAATGCCGTCAAAA-3′), EREG 5′-TCCCAGGAGAG TCCAGTGAT-3′ and 5′-GTGTTCACATCGGACACCAG-3′, AREG (5′-CCACAGTGCTGATGGATTTG-3′ and 5′-GCCAGGTATTTGTGGTTCGT-3′), CYR61 (5′-ATGAATTGATTGCAGTTGGAAA-3′ and 5′-TAAAGGGTTGTATAGGATGCGA-3′), RNA18S (5′-TGTGGTGTTGAGGAAAGCAG-3′ and 5′-TCCAG ACCATTGGCTAGGAC-3′), CCND1 (5′-GCTGTGCATCTACACCGACA-3′ and 5′-TTGAGCTTGTTCACC AGGAG-3′) and MYC (5′-CTTGTTGCGGAAACGACGAG-3′ and 5′-ACTCAGCCAAGGTTGTGAGG-3′). Target cDNA expression was quantified using the comparative ΔΔCt method with 18S rRNA as an internal control.

## 3. Statistical Analysis

### 3.1. Descriptive and Univariate Analyses

Quantitative variables are presented as their mean and standard deviations and compared to univariate analyses with a Student’s t-test if following a Gaussian distribution (Shapiro–Wilk tests were used to assess the Gaussian distribution) or a Wilcoxon’s rank test if non-Gaussian distribution. Similarly, the qualitative variables are described by their absolute numbers and percentages, and are subsequently compared using Pearson’s χ^2^ test if effectives were sufficient or by a Fisher’s exact test if not. In Table S2, the *p*-value refers to the relationship between Cav1 expression subgroups and the characteristics of the patients.

### 3.2. Survival Analyses

Overall survival (OS) and locoregional recurrence-free survival were estimated using the Kaplan–Meier method. Inferential analysis for qualitative variables was performed using a log-rank test, and quantitative variables were compared using the Cox model. Multivariate analyses were performed using all statistically significant variables in univariate analyses or according to clinical importance. A stepwise regression was performed with backward selection to identify variables of potential prognostic relevance. *p* < 0.05 was considered significant. All analyses were performed with R 3.1.0 software (https://cran.r-project.org, access on 3 May 2021) and the survival package.

### 3.3. Measures Interrater Agreement

The degree of agreement between the two pathologists was estimated using the weighted Cohen’s kappa coefficient (k).

## 4. Results

### 4.1. Overexpression of Cav1 Enables Cells to Survive Long-Term Exposure to Cetuximab Alone and in Combination with Radiation

The basal subtype represents 30% of HNSCC tumors and was previously described as being more sensitive to EGFR-targeting treatments [30,31]. We chose SCC9 and CAL33 cell lines as representative of this subtype [32] to determine the impact of Cav1 on the response to cetuximab. Cells were manipulated to overexpress Cav1 and were exposed to cetuximab (CTX). 

**Figure 1:**
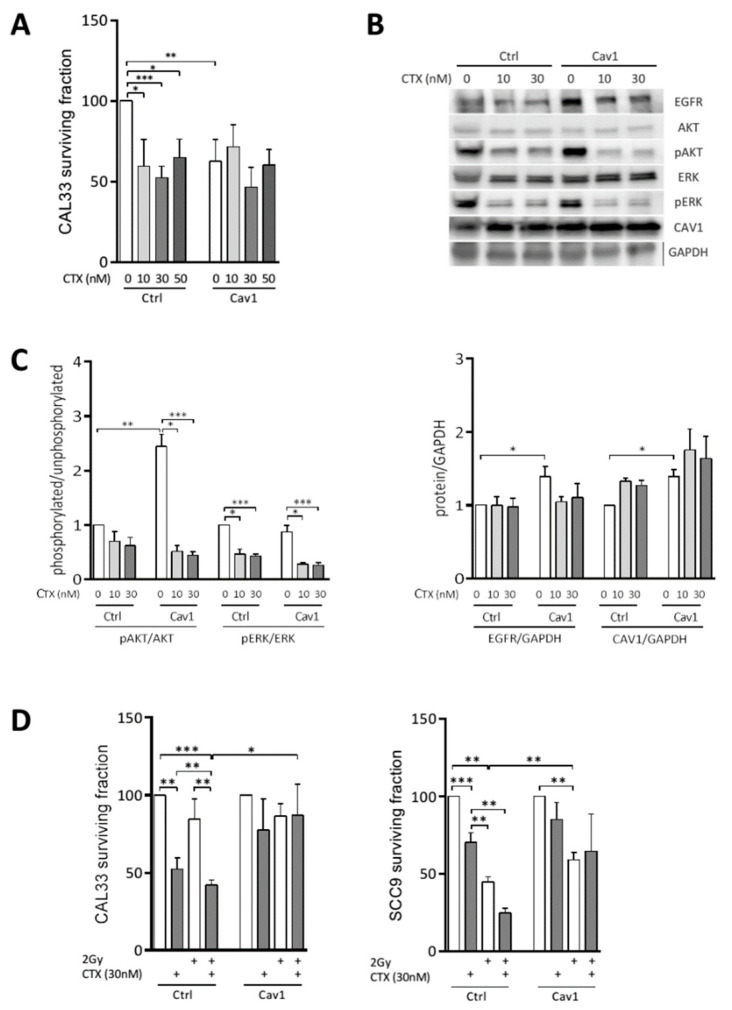
The overexpression of Cav1 enables cells to survive long-term exposure to CTX alone and in combination with radiation. (**A**) Clonogenic survival of CAL33 cells transfected with pEZ-M68_ctrl_ (CAL33_Ctrl_) or pEZ-M68_cav1_ (CAL33_Cav1_) was determined 10 days after exposure to 10, 30 and 50 nM CTX. Histograms represent the surviving fraction in each condition. The plating efficiencies were 0.21 ± 0.07 and 0.16 ± 0.04 for CAL33_Ctrl_ and CAL33_Cav1,_ respectively. Data are represented as the mean (*n* = 3–5) ± SEM (* *p* < 0.05, ** *p* < 0.01 and *** *p* < 0.001). (**B**) Expression of EGFR, AKT, phospho-AKT, ERK1/2, phospho-ERK1/2 and GAPDH was determined by Western blot (WB) of lysates from CAL33_Ctrl_ and CAL33_Cav1_ cells exposed 72 h to 0, 10 and 30 nM CTX. Western blots are representative of 3-5 independent experiments. (**C**) Phospho-AKT, phospho-ERK, EGFR and Cav1 levels were analyzed using AKT, ERK or GAPDH as a loading control. Each bar represents the mean (*n* = 3–5) ± SEM (* *p* < 0.05, ** *p* < 0.01 and *** *p* < 0.001). (**D**) Clonogenic survival of SCC9_Ctrl_, SCC9_Cav1,_ CAL33_Ctrl_ and CAL33_Cav1_ was determined 10 days after exposure to 2 Gy irradiation, 30 nM CTX and a combination of both. Histograms represent the surviving fraction in each condition. Data are represented as the mean (*n* = 3–5) ± SEM (* *p* < 0.05, ** *p* < 0.01 and *** *p* < 0.001).

CTX significantly reduced the long-term clonogenic survival of the CAL33_Ctrl_ cell line at all concentrations tested (41 ± 16%, 48 ± 7% and 35 ± 11% at 10, 30 and 50 nM, respectively, Figure 1A). The overexpression of Cav1 significantly reduced basal clonogenic survival. Nevertheless, it rendered CAL33_Cav1_ cells totally resistant to CTX (Figure 1A). Similar results were observed in SCC9_ctrl_ and SCC9_Cav1_ cells. We next determined whether the resistance was linked to the inhibition of EGFR expression or its downstream signaling pathway. Cav1 overexpression increased the steady-state expression of EGFR, and was associated with increased phosphorylation of AKT (Figure 1B,C). CTX significantly inhibited ERK1/2 phosphorylation in CAL33_Ctrl_ and CAL33_Cav1_. Although it did not affect the phosphorylation of AKT in CAL33_Ctrl_, CTX significantly inhibited AKT phosphorylation in CAL33_Cav1_ (Figure 1B,C). EGFR and Cav1 expression remain unaffected by cetuximab. The data show that CTX blocked AKT and ERK1/2 activity in CAL33_Ctrl_ and CAL33_Cav1_, indicating that these pathways remain sensitive to CTX but play a minor role in the resistance of Cav1-overexpressing cells to CTX.

SCC9 and CAL33 cells were exposed to 30 nM CTX before being irradiated with 2 Gy. In CAL33_Ctrl_, the combination of CTX with irradiation reduced clonogenic survival by more than 61 ± 2%, which is more than that obtained by each treatment alone (48 ± 7% and 16 ± 13% reduction with CTX or irradiation alone, respectively, Figure 1D left). In contrast, clonogenic survival was not affected regardless of CAL33_Cav1_ exposure. We confirmed these results in another representative cell line of the basal subtype exerting less intrinsic sensitivity to CTX but higher radiosensitivity—SCC9. Almost similar data were obtained with SCC9_Ctrl_. The clonogenic survival was reduced by more than 73 ± 4% following exposure to CTX + irradiation vs. 30 ± 6% and 55 ± 3% reductions with CTX and irradiation alone, respectively (Figure 1D right). The CTX-induced inhibition of clonogenic survival and radiosensitization was abolished in SCC9_Cav1_ cells. Cav1 overexpression did not modify the intrinsic radiosensitivity of any of the cells tested (not shown). In conclusion, Cav1 overexpression reduces the intrinsic clonogenicity of HNSCC cells but also triggers cell resistance to conventional CTX-radiotherapy regimens.

### 4.2. Overexpression of Cav1 Protects Cells Against the Cytostatic Effect of Cetuximab Alone or Combined with Irradiation

**Figure 2:**
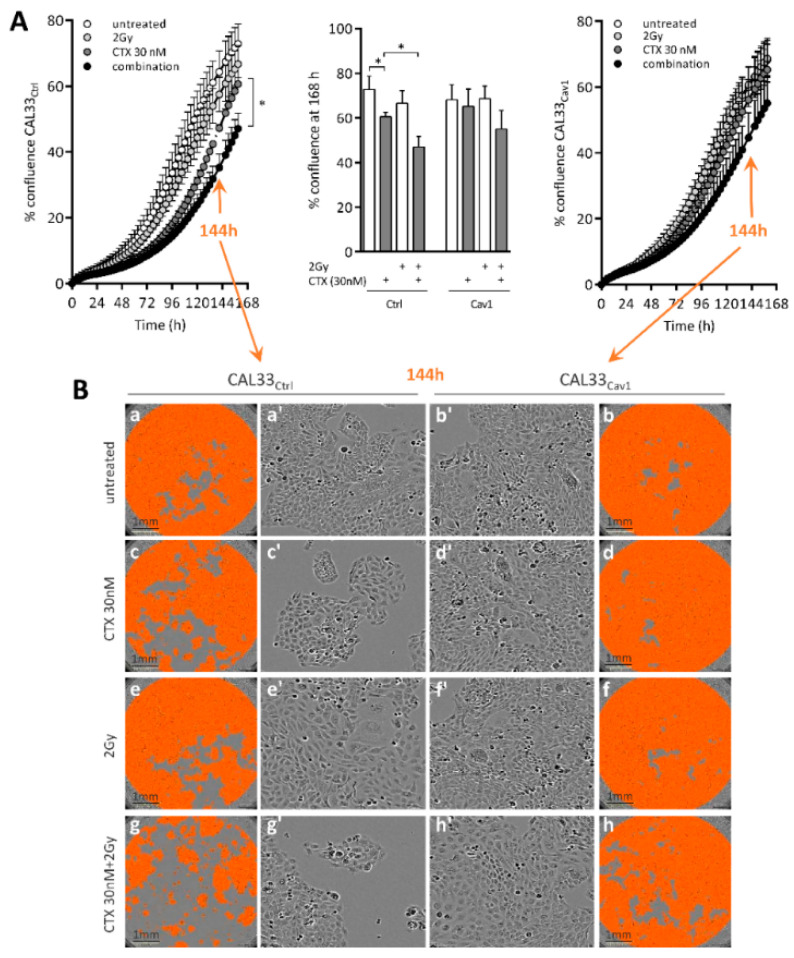

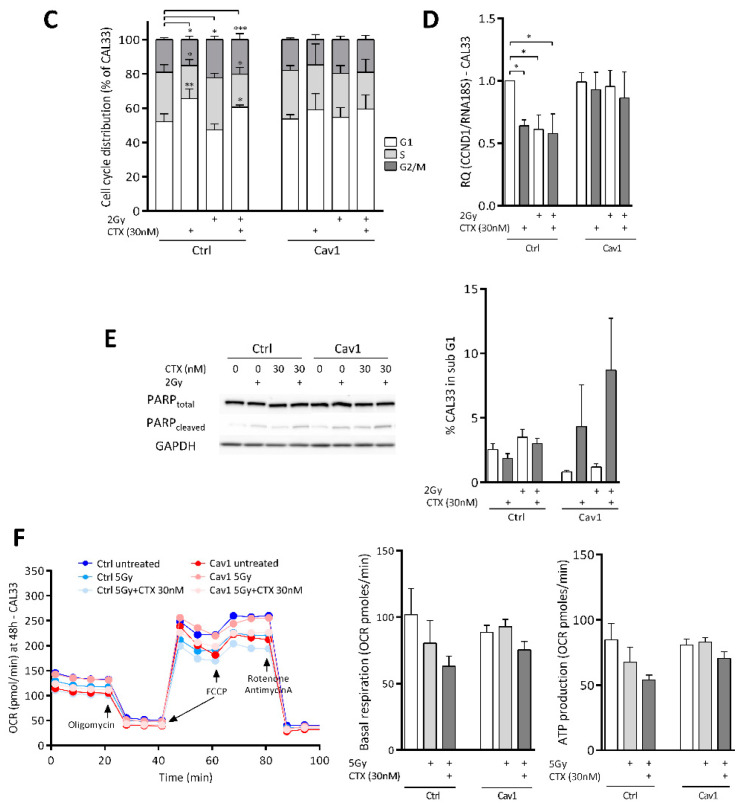
The overexpression of Cav1 protects cells against the cytostatic effect of CTX alone or combined with irradiation. (**A**) Growth was monitored by analyzing the area occupied by cells (% confluence) over 7 days (168 h) using the Incucyte^®^ Zoom (Sartorius, Goettingen, Germany). Curves show the % confluence (after normalization to day 0) of CAL33_Ctrl_ (left) and CAL33_Cav1_ (right) cells over 7 days (168 h) of exposure to 2 Gy irradiation, 30 nM CTX, or a combination of both. Histograms represent the % confluence at day 7 (168 h) for each condition. Data are represented as the mean (*n* = 3–5) ± SEM (* *p* < 0.05). (**B**) IncuCyte imaging showing the confluence of the cells (represented by the orange mask, a–h) and the morphology of cells (a’–h’) at day 6 (144 h). (**C**), the progression of the cell cycle was determined 48 h after exposure to 2 Gy irradiation, 30 nM CTX, and a combination of both by flow cytometry. Histograms represent the percentage of cells in each phase of the cell cycle (G1, S and G2/M). Data are represented as the mean (*n* = 3–5) ± SEM (* *p* < 0.05, ** *p* < 0.01 and *** *p* < 0.001). (**D**) Quantitative determination of CCND1 transcripts in CAL33_Ctrl_ and CAL33_Cav1_ cells exposed for 72 h to 2 Gy irradiation, 30 nM CTX, or a combination of both using RT-qPCR with RNA18S as a control. Each bar represents the mean (*n* = 3–5) ± SEM (* *p* < 0.05). (**E**) CAL33_Ctrl_ and CAL33_Cav1_ cells were exposed for 48 h to 2 Gy irradiation, 30 nM CTX and a combination of both. Expression of cleaved and total PARP and GAPDH was determined by Western blot (left). Apoptosis was determined by flow cytometry (right). Histograms represent the percentage of cells in subG1. Data are represented as the mean (*n* = 3–5). (**F**) OCR and ECAR were measured using the mitochondrial stress test procedure in CAL33_Ctrl_ and CAL33_Cav1_ cells 7 days after treatment with 2 Gy irradiation alone or combined with 30 nM CTX. Basal respiration (last rate measurement before oligomycin injection—nonmitochondrial respiration), maximal respiration (maximum rate after FCCP injection—nonmitochondrial respiration) and ATP production (last rate measurement before oligomycin injection—minimum rate measurement after oligomycin injection) were determined. Histograms represent the mean (*n* = 3) ± SEM.

To determine which processes sustain the survival capacities conferred by Cav1, growth (monitored by analyzing the area occupied by cells (% confluence) over time), cell health and morphology were monitored for 7 days (168 h) using a real-time live-cell bioimager. Despite the fact that Cav1 overexpression reduced basal clonogenicity (Figure 1A), CAL33_Cav1_ cells filled the growing area at the same rate as CAL33_Ctrl_ cells (Figure 2A curves and histograms and B a and b). The growth of CAL33_Ctrl_ was significantly reduced by 14% with CTX and by 26% with CTX combined with irradiation (Figure 2A left and histograms and Figure 2B c–g). Growth was not altered in any condition tested in CAL33_Cav1_ cells (Figure 2A right and histograms, and Figure 2B d–h). No modification of the size or shape of the cells could be observed during the course of the experiment (Figure 2B a’–h’).

As expected from the clonogenic assay and growth data, the distribution of CAL33_Ctrl_ cells in the cell cycle was significantly altered upon treatment (Figure 2C). Indeed, CTX increased the percentage of cells in G1. Irradiation arrested cells in G2/M. The combination of both treatments blocked cells in G1 and G2/M (Figure 2C). Cell cycle arrest in G1/S was associated with a reduction in CCDN1 expression (Figure 2D). Regardless of the treatment used, no alteration in cell cycle progression or the expression of CCND1 was observed in CAL33_Cav1_ cells (Figure 2C,D). No apoptosis could be measured (by PARP cleavage or flow cytometry, Figure 2E) in any of the conditions tested. As mitochondria might influence the therapeutic response and play a critical role in the regulation of cell survival triggered by cancer treatment, we checked the integrity of the mitochondria. In contrast to CAL33_Cav1_ cells, in which both parameters remained unaffected, basal respiration and ATP production were reduced in CAL33_Ctrl_ cells exposed to CTX alone or in combination with irradiation for 48 h (Figure 2F). Thus, protection of the mitochondria might contribute to the resistance induced by Cav1. The data suggest that Cav1 enables cells to survive long-term exposure to CTX alone or combined with radiation at least in part by maintaining growth, cell cycle progression and mitochondrial integrity, altogether contributing to protecting cells against death.

### 4.3. Overexpression of Cav1 Maintains the Evasive Potency of Cells Exposed to Cetuximab Alone or Combined with Irradiation

**Figure 3:**
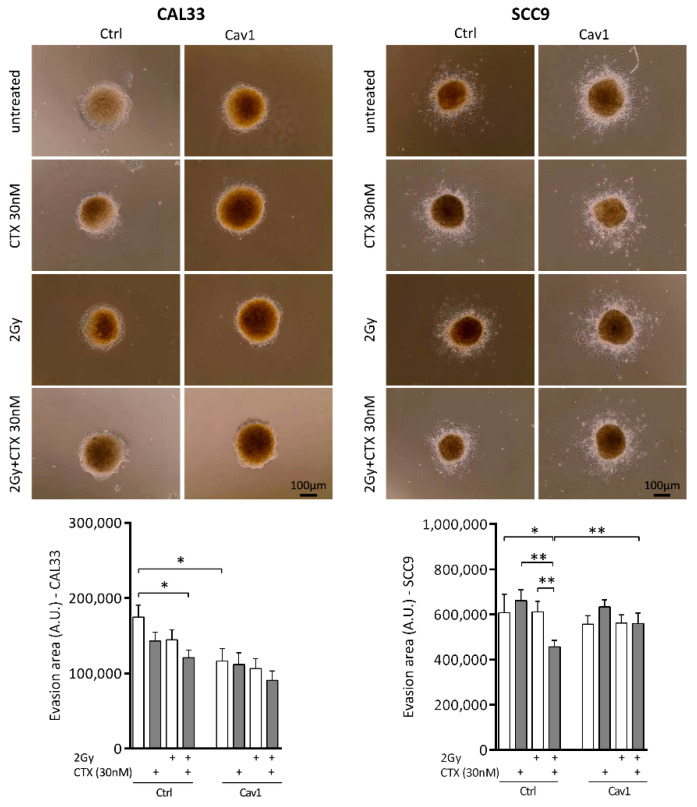
Overexpression of Cav1 maintains the evasive capacity of cells exposed to CTX alone or combined with irradiation. Analysis of collective cell migration: evasion of SCC9_Ctrl_, SCC9_Cav1,_ CAL33_Ctrl_ and CAL33_Cav1_ cells 24 h after treatment with 2 Gy irradiation, 30 nM cetuximab and a combination of both out of the spheroids. Pictures show the evasion area and histograms represent the mean ± SEM area covered by cells escaping from the spheroid (*n* = 3–4, * *p* < 0.05, and ** *p* < 0.01).

To evaluate the impact of the deregulation of Cav1 expression on the propensity of tumor cells to evade the primary tumor sphere and thus to colonize tumor surroundings, 3D collective cell migration assays were performed using CAL33 spheroids. Although CTX and irradiation by themselves did not significantly affect the evasion capacity of CAL33_Ctrl_ cells, the combination of both reduced evasion out of the tumor spheroid by 31% (Figure 3 left). The overexpression of Cav1 reduced basal evasive capacity by 33% (Figure 3 left). CTX, irradiation, or the combination of both, did not inhibit evasion any further (Figure 3 left). Similar results were observed in the other basal-like cell line SCC9 (Figure 3, right). Even if CTX and irradiation by themselves did not significantly affect the evasive capacity of SCC9_Ctrl_ cells, the combination of both treatments reduced the evasion out of the tumor spheroid by 25% (Figure 3 right). The overexpression of Cav1 affected neither basal evasion nor the motility of cells exposed to CTX, irradiation, or the combination of both (Figure 3 right).

Thus, in addition to survival and growth preservation, Cav1 overexpression maintains the evasive capacities of cells exposed to CTX-radiotherapy.

### 4.4. Overexpression of Cav1 Is Associated with a Decrease in EREG-Driven Oncogenic Addiction

**Figure 4:**
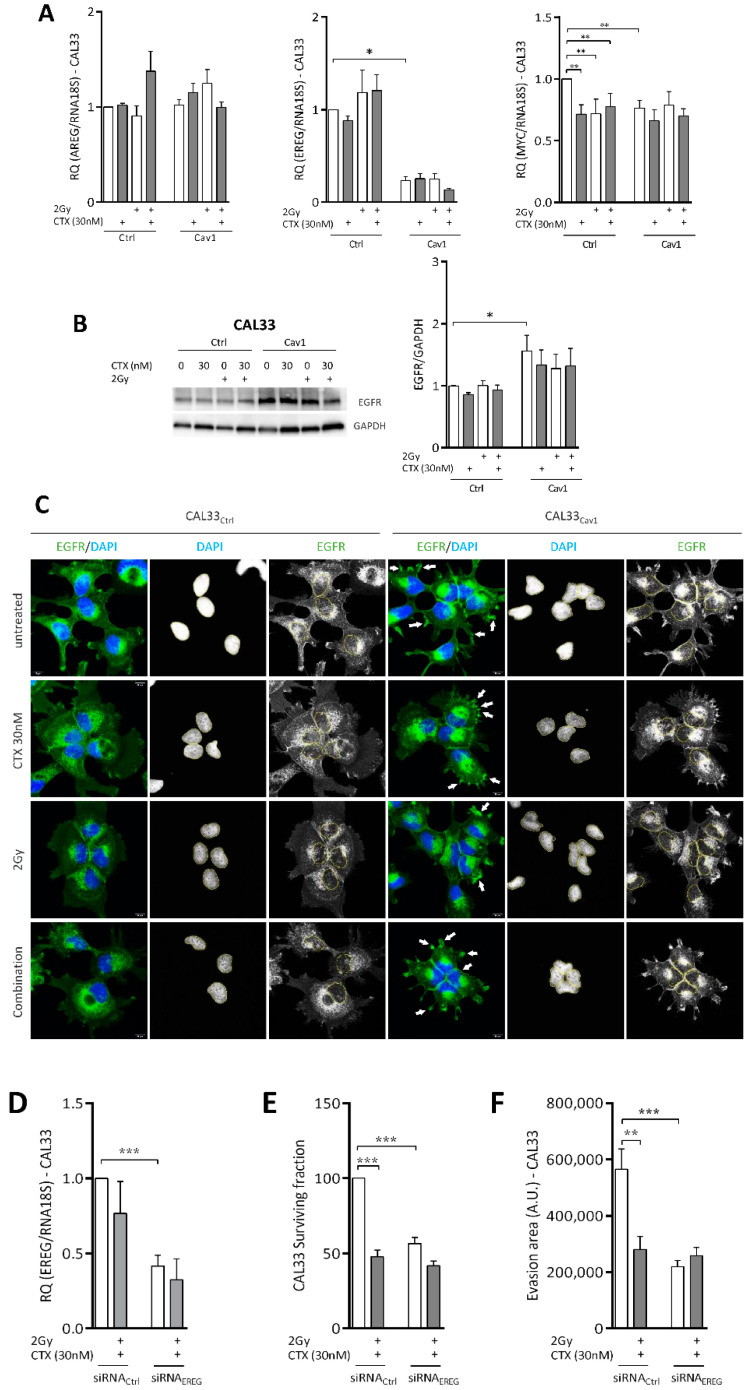
Overexpression of Cav1 is associated with a decrease in EREG-driven oncogenic addiction. (**A**) Quantitative determination of AREG, EREG and MYC transcripts in CAL33_Ctrl_ and CAL33_Cav1_ cells exposed to 2 Gy irradiation, 30 nM cetuximab or a combination of both using RT-qPCR with RNA18S as a control. Each bar represents the mean (*n* = 3–5) ± SEM (* *p* < 0.05, ** *p* < 0.01). (**B**) Expression of EGFR and GAPDH was determined by Western blot (WB) in CAL33_Ctrl_ and CAL33_Cav1_ cells exposed to 2 Gy irradiation, 30 nM cetuximab and a combination of both. Western blots are representative of 3–5 independent experiments. EGFR levels were analyzed using GAPDH as a loading control. Each bar represents the mean (*n* = 3-5) ± SEM (* *p* < 0.05). (**C**) Immunofluorescence analysis of EGFR by confocal microscopy in CAL33_Ctrl_ and CAL33_Cav1_ cells exposed to 2 Gy irradiation, 30 nM CTX, and a combination of both. Nuclei were stained using DAPI. Arrows show the region of the plasma membrane concentrating EGFR staining (× 63). (**D**) Quantitative determination of EREG transcripts in CAL33_siRNA-Ctrl_ and CAL33_siRNA-EREG_ cells exposed to a combination of 2 Gy irradiation and 30 nM CTX using RT-qPCR with RNA18S as a control. Each bar represents the mean (*n* = 3–5) ± SEM (*** *p* < 0.001). (**E**) Clonogenic survival of CAL33_siRNA-Ctrl_ and CAL33_siRNA-EREG_ cells was determined 10 days after exposure to a combination of 2 Gy irradiation and 30 nM CTX. Histograms represent the surviving fraction in each condition. Data are represented as the mean (*n* = 3–5) ± SEM (*** *p* < 0.001). (**F**) Analysis of collective cell migration: evasion of CAL33_siRNA-Ctrl_ and CAL33_siRNA-EREG_ 24 h after treatment with a combination of 2 Gy irradiation and 30 nM CTX out of the spheroids. Pictures show the evasion area and histograms represent the mean (*n* = 3–4) ± SEM (** *p* < 0.01 and *** *p* < 0.001) area covered by cells evading the spheroid.

The basal subtype of HNSCC tumors aberrantly expresses factors involved in EGFR signaling, such as amphiregulin (AREG), epiregulin (EREG), CAIX oand HIF1A [33,34,35,36]. In accordance with this, we showed that representative cells of this subtype exert higher expressions of AREG and EREG than nonbasal cell subtypes. The expression of EREG was found to be a predictive functional marker of sensitivity to EGFR blockade in basal-like HNSCC [32]. The exposure of CAL33_ctrl_ and CAL33_Cav1_ cells to CTX, irradiation, or the combination of both did not affect the expression of AREG and EREG (Figure 4A). However, the overexpression of Cav1 almost totally abolished EREG expression (77 ± 4%, Figure 4A) without AREG being affected. As observed for EREG, the expression of another EGFR target gene, MYC, was also significantly reduced by CTX, irradiation, or the combination of both in CAL33_Ctrl_ cells (Figure 4A). As observed for EREG, Cav1 overexpression was associated with an inhibition of its expression (24 ± 6%, Figure 4A) that was not further affected by any treatment. Altogether, the data suggest that the expression of Cav1 might render cells insensitive to EGFR signaling at least in part by preventing the addictive EREG feedback loop.

EGFR expression was increased in CAL33_Cav1_ cells (Figure 4B). It remained unaffected by CTX, irradiation or the combination of both (Figure 4B). Immunofluorescence staining followed by confocal analysis showed that EGFR was variably distributed in CAL33_Ctrl_ and CAL33_Cav1_ cells. EGFR is mainly located at the plasma membrane and in the cytosol of CAL33_Ctrl_ cells. In contrast, EGFR seemed to concentrate into clusters at specific regions of the plasma membrane and around the nucleus in CAL33_Cav1_ cells (see arrows Figure 4C). Nuclear staining was observed in both cell types in the resting state (Figure 4C). The exposure of CAL33_Ctrl_ cells to CTX, irradiation or a combination of both causes internalization of EGFR, characterized by the accumulation of endocytic vesicles in the cytosol. In addition, increased EGFR staining could be detected in the nucleus, most likely due to its nuclear translocation. No change in EGFR distribution could be observed in CAL33_Cav1_ cells regardless of the treatment used (Figure 4C). The data suggest that the overexpression of Cav1 not only suppresses EREG, but also redistributes and anchors EGFR in specific regions, thus preventing its internalization.

EREG was silenced in CAL33_Ctrl_ cells (using a siRNA, CAL33_siRNA-EREG_) to determine its role in the resistance phenotype of CAL33_Cav1_ cells. EREG expression was repressed by 59 ± 7%, and was not decreased any further by the combination of CTX and irradiation (Figure 4D). The downregulation of EREG reduced clonogenic survival to a similar extent to that obtained following Cav1 overexpression (47 ± 2% reduction Figure 4E vs. 38% ± 13% Figure 1A). Exposure to CTX and irradiation reduced the clonogenic survival of CAL33_siRNA-Ctrl_ by more than 57 ± 4%, and only mildly affected that of CAL33_siRNA-EREG_, i.e., 16 ± 3% (Figure 4E). Turning to evasion, the downregulation of EREG reduced basal evasive capacity by 61% (Figure 4F). Exposure to CTX and irradiation reduced the evasion of CAL33_siRNA-Ctrl_ by more than 51%, and had no effect on CAL33 _siRNA-EREG_ (Figure 4F). Notably, Cav1 expression was affected neither by the downregulation of EREG nor by the treatment (not shown). Together, the data confirm that the repression of EREG recapitulates the phenotype of Cav1-overexpressing cells, and suggest that EREG is a key factor in the chemo/radioresistant phenotype of Cav1-expressing cells.

### 4.5. Repression of EREG by Cav1 Is Associated With the Activation of the Hippo-YAP Pathway

**Figure 5:**
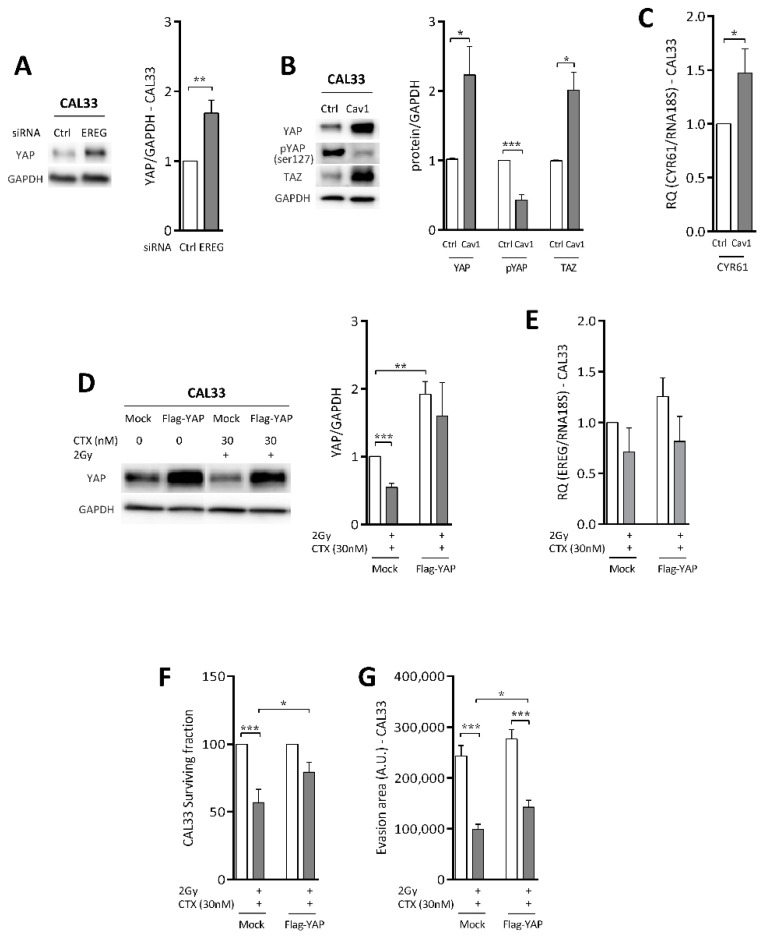
Repression of EREG by Cav1 is associated with the activation of the HIPPO-YAP pathway. (**A**) Expression of YAP and GAPDH was determined by Western blot (WB) in CAL33_siRNA-Ctrl_ and CAL33_siRNA-EREG_ cells. Western blots are representative of 3–5 independent experiments. YAP levels were analyzed using GAPDH as a loading control. Each bar represents the mean (*n* = 3–5) ± SEM (** *p* < 0.01). (**B**) Expression of YAP, phospho-YAP, TAZ and GAPDH was determined by the Western blotting (WB) of lysates from the CAL33_Ctrl_ and CAL33_Cav1_ cells. Western blots are representative of 3–5 independent experiments. YAP levels were analyzed using GAPDH as a loading control. Each bar represents the mean (*n* = 3–5) ± SEM (* *p* < 0.05 and *** *p* < 0.001). (**C**) Quantitative determination of transcripts of CYR61 in CAL33_Ctrl_ and CAL33_Cav1_ cells using RT-qPCR with RNA18S as control. Each bar represents the mean (*n* = 3-5) ± SEM (* *p* < 0.05). (**D**) The expression of YAP and GAPDH was determined by Western blot (WB) in CAL33_Mock_ and CAL33_Flag-YAP_ cells after treatment with a combination of 2 Gy irradiation and 30 nM CTX. Western blots are representative of 3–5 independent experiments. YAP levels were analyzed using GAPDH as a loading control. Each bar represents the mean (*n* = 3–5) ± SEM (** *p* < 0.01 and *** *p* < 0.001). (**E**) Quantitative determination of EREG transcripts in CAL33_Mock_ and CAL33_Flag-YAP_ after treatment with a combination of 2Gy irradiation and 30 nM CTX using RT-qPCR with RNA18S as a control. Each bar represents the mean (*n* = 3–5) ± SEM. (**F**) Clonogenic survival of CAL33_Mock_ and CAL33_Flag-YAP_ cells was determined 10 days after exposure to a combination of 2 Gy irradiation and 30 nM CTX. Histograms represent the surviving fraction in each condition. Data are represented as the mean (*n* = 3–5) ± SEM (* *p* < 0.05 and *** *p* < 0.001). (**G**) Analysis of collective cell migration: evasion of CAL33_Mock_ and CAL33_Flag-YAP_ cells 24 h after treatment with a combination of 2 Gy irradiation and 30 nM CTX out of the spheroids. Pictures show the evasion area and histograms represent the mean ± SEM area covered by cells evading the spheroid (*n* = 3–4, * *p* < 0.05 and *** *p* < 0.001).

YAP expression was recently shown to be associated with the resistance of colorectal cells to CTX [37]. In addition, EGFR and the Hippo-YAP pathway seem to regulate each other. Here, the downregulation of EREG significantly induced YAP expression (1.7 ± 0.2%, Figure 5A). In accordance with this, the overexpression of Cav1, which represses EREG, was also associated with the induction of YAP and its paralog TAZ (Figure 5B, 2.2 ± 0.4- and 2.0 ± 0.2-fold increase in CAL33_Cav1_ versus CAL33_Ctrl_). Induction of YAP expression was associated with an inhibition of its phosphorylation at Ser127 by 57 ± 7% in CAL33_Cav1_ cells, suggesting an increase in YAP activity in Cav1-overexpressing cells (Figure 5B). This was confirmed by the induction of the target-gene of YAP, CYR61 (Figure 5C).

Next, YAP was overexpressed in CAL33_Ctrl_ cells (CAL33_Flag-YAP_) to determine its role in the resistant phenotype of CAL33_Cav1_. YAP expression was increased 1.9 ± 0.2-fold (Figure 5D). Exposure to CTX and irradiation reduced the expression of YAP in CAL33_Mock_ cells (46 ± 6%), but remained unaffected in CAL33_Flag-YAP_ cells (Figure 5D). Of interest, the expression of YAP did not modify the levels of EREG (Figure 5E). In contrast to Cav1 and EREG, the forced expression of YAP did not affect basal clonogenic survival. Exposure to CTX and irradiation reduced the clonogenic survival of CAL33_Mock_ by more than 44 ± 10% without affecting that of CAL33_Flag-YAP_ (Figure 5F). The overexpression of YAP did not affect basal evasion (Figure 5G). Exposure to CTX and irradiation reduced the motility of CAL33_Mock_ and CAL33_Flag-YAP_ to similar extents (59% and 49%, respectively, Figure 5G). Similar results were obtained using the constitutively active mutant form of YAP, S127A (not shown). Again, Cav1 expression was affected neither by the expression of YAP nor by the treatment (not shown). Altogether, data show that the expression of YAP only partially recapitulates the phenotype of Cav1-overexpressing cells. Although YAP did not modify basal survival and motility, it prevented the damage of the treatment.

### 4.6. Tumors Overexpressing Cav1 Give Rise to Locoregional Relapse and Have a Poor Prognosis

**Figure 6:**
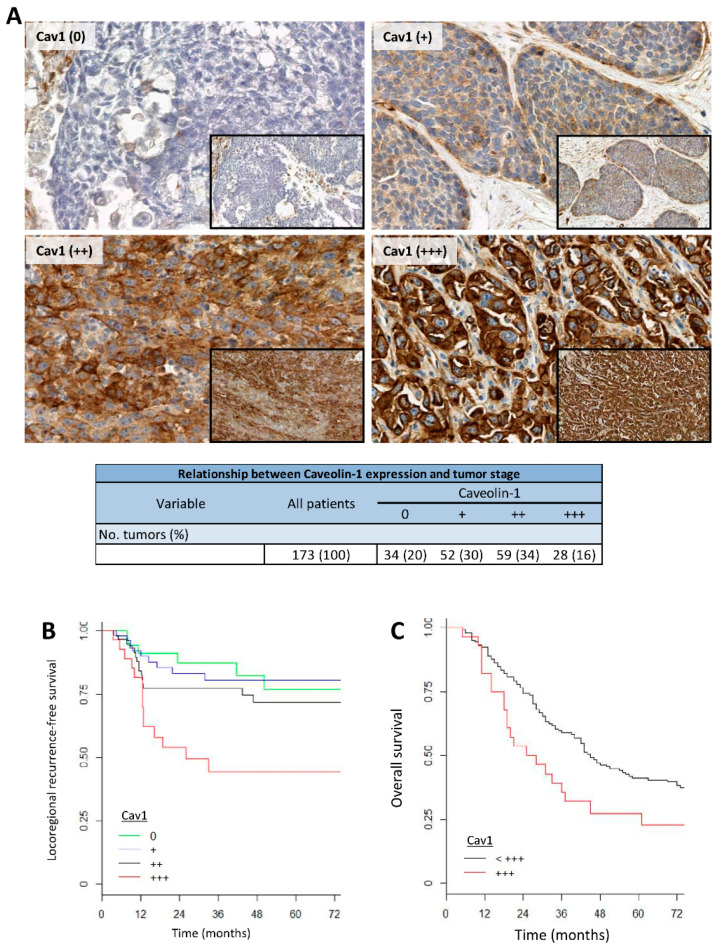
Cav1 expression in human HNSCC primary tumor tissues. (**A**) Semiquantitative analysis of the immunohistochemical staining of Cav1 based on classification in 4 categories according to the percentage of Cav1-positive carcinoma cells: 0: 0%; +: 1–25%; ++: 26–75%; +++: > 75% (original magnification: × 10 and × 40). (**B**) Locoregional recurrence-free survival. (**C**) Overall survival was analyzed according to Cav1 protein expression using the Kaplan–Meier estimate with the log rank test.

We showed that Cav1 confers resistance, allowing cells to survive, proliferate or escape tumor spheroids even under extreme conditions, such as the CTX-radiotherapy regimens used in the clinical management of LA-HNSCC. It was tempting to speculate that Cav1 might play a role in the recurrence of these tumors. One hundred seventy-three patients with stage III–IVb primary HNSCC (median age: 58 years, range 36–84 years) were enrolled in this study. Nearly all patients received post-operative radiotherapy (98%) or chemoradiotherapy (cisplatin, see Table S2 for the baseline patient characteristics). Immunoreactivity indicating the expression of Cav1 was detected both in the cytoplasm and at the cell membrane (Figure 6A).

Thirty-four tumors (20%) did not express Cav1 (Cav1 (0)). The remaining 80% expressed Cav1 at different intensities: 30% (52), 34% (59) and 16% (28) of the samples displayed low (Cav1 (+)), intermediate (Cav1 (++)) and high (Cav1 (+++)) Cav1 staining (Table in Figure 6A). The H-Scores for each subgroup were 24.8 ± 5.9 for Cav1 (0), 76.4 ± 7.5 for Cav1 (+), 156.8 ± 9.7 for Cav1 (++) and 223.2 ± 12.1 for Cav1 (+++) (Data S1). The Cav1 H-Scores were significantly different between each group, which validates the robustness of our subgrouping. The expression of CAV1 was also assessed by RT-qPCR. CAV1 gene expression for each subgroup was 75.0 ± 22.7 for Cav1 (0) samples, 89.9 ± 8.2 for Cav1 (+) samples, 194.8 ± 24.4 for Cav1 (++) samples and 325.4 ± 65.5 for Cav1 (+++) samples (Data S2). Again, CAV1 gene expression could statistically discriminate each subgroup. In addition, CAV1 gene expression was correlated with the IHC expression of the protein (*p* < 0.0001, Data S3).

Cav1 expression (subgroups, H-score or mRNA) was not correlated with tumor differentiation, age, sex, tumor stage (T1 to T4) or lymph node status (*p* > 0.05). The high IHC expression of Cav1 was significantly associated with an increased risk of local recurrence (*p* = 0.00124, HR = 3.9 (1.5; 10.0) and H-score *p*-value of 0.01364) (Figure 6B). High CAV1 gene expression was also correlated with a higher risk of local recurrence (*p* = 0.013). Finally, a strong expression of Cav1 (+++) was associated with shorter overall survival (*p* = 0.02) (Figure 6C). Altogether, the data suggest that Cav1 expression is correlated with tumor recurrence and poor prognosis.

## 5. Discussion

The biphasic expression of Cav1 has already been described in HNSCC [15,38]. Cav1 gradually increases during epithelial transformation into primary SCC and decreases afterwards in metastatic carcinoma. Cav1 expression was also reported to be induced by various drugs (metformin, cisplatin) [25] as well as by irradiation [39]. Zhang and colleagues reported a loss of Cav1 in highly metastatic cells. The restoration of Cav1 in these cells completely abolished metastasis [15]. In addition, we reported low or no expression of Cav1 in metastatic-prone primary tumors [13]. The deletion of Cav1 in HNSCC cell lines boosted cell motility through the activation of α_5_β_1_ integrins and MMPs [13]. By contrast, Masuelli and colleagues [24] suggested that the overexpression of Cav1 and ErbB receptors, including EGFR, promoted metastasis. The present study showed that the overexpression of Cav1 is associated with locoregional relapse and poor prognosis. Tumor recurrence is probably linked to the fact that Cav1 maintains/preserves the growth, survival and motility of cells exposed to CTX-radiotherapy. As Cav1 can alter the therapeutic response of tumors, thereby increasing the likelihood of recurrence, it should be considered not only as a biomarker predicting recurrence, but also as a marker predicting therapeutic failure. Although cisplatin delivered concurrently with radiotherapy is the SOC in LA-HNSCC, CTX represents the main alternative agent to definitive radiotherapy for cisplatin-ineligible LA-HNSCC patients. There are no data available on the proportion of cisplatin-ineligible LA-HNSCC patients, but this proportion is probably significant, with a rough estimate based on the proportion of recurrent or metastatic HNSCC patients treated with carboplatin in the two pivotal trials in first-line treatment (EXTREME study and KEYNOTE-048) in the range of 40 to 50% [4,8,40,41]. Although it would have been interesting and appropriate to include study arms with cisplatin, we wanted in this paper to focus on this targeted therapy to investigate resistance mechanism in HNSCC cancer. As observed in other cancers, HNSCC presents intrinsic or therapeutically acquired resistance to CTX. This can primarily be attributed to a deregulation of EGFR itself (overexpression and strong activity in more than 80% of HNSCC) [42,43], the expression of a mutant type III variant of EGFR [44,45], aberrant downstream signaling pathways [46,47,48], an oncogenic switch [49,50,51,52] or compensatory mechanisms (such as the activity of other tyrosine-kinase receptor ALK [53], MET [54,55] or AXL [56,57]). CTX even seems to promote its own resistance by inducing mutations in KRAS/NRAS/HRAS [58] or missense mutations in the ectodomain of EGFR, mimicking the activation of the receptor [59]. In this study, we reported that Cav1 may also play a role in this process. The overexpression of Cav1 not only abolished the sensitivity to CTX but also prevented radiosensitization. Cav1-expressing cells exert better survival capacities and remain proliferative and motile when exposed to CTX-radiotherapy. Historically, Cav1 was reported to be a negative regulator of various tyrosine-kinase receptors, including EGFR [16]. Cav1 was reported to block EGF-mediated proliferation, migration and invasion by targeting downstream effectors (mainly MEK/ERK and Pi3K/AKT/mTor) in various cancers [60,61,62,63]. However, Cav1 can also promote the interaction between EGFR and its effectors, leading to signal transduction in caveolae [20,24,64]. Bound to Cavin via ROR1, Cav1 allows EGFR/Met/IGFR signaling and subsequent resistance to anti-EGFR and TKIs [28,65]. Although EGFR, AKT and ERK1/2 expression and/or activity are increased in Cav1-expressing cells, CTX was still able to reduce their phosphorylation. Thus, unregulated EGFR signaling cannot account for the resistance of Cav1-overexpressing cells.

Cav1 also plays a crucial role in EGFR trafficking. In breast cancer cells, it internalizes HER2 bound to trastuzumab, which improves drug efficacy [21,25,27]. In contrast, knocking out Cav1 in gastric cancer cells increases the availability of HER2 at the cell surface, thus sensitizing gastric cancer cells to anti-HER2 therapy [66]. However, EGFR can also signal far away from the plasma membrane. After irradiation, Cav1 translocates EGFR to the nucleus where it activates DNA-PK and DNA repair [22,23,67]. EGFR also exerts transcriptional activity that is manifested by the upregulation of CCND1 or MYC, supporting EGFR-mediated proliferation/survival [18]. Here, we have shown that EGFR concentrates into clusters at specific regions of the plasma membrane in Cav1-expressing cells. EGFR remained blocked there after exposure of the cells to CTX and/or irradiation, even though it was internalized in control cells. The concentration of EGFR in lipid rafts by Cav1 was reported to negatively regulate EGFR signaling [19]. It could also prevent EGFR internalization and subsequent nuclear transcriptional activity. In accordance, the expression of the EGFR target-gene MYC and the EGFR ligand EREG were inhibited in Cav1-expressing cells. Epiregulin, encoded by the EREG gene, binds to and activates EGFR and ErbB4/HER4 [68]. Job and colleagues recently suggested that the sensitivity of basal-like HNSCC to EGFR-targeting drugs comes from their addiction to EGFR signaling through an oncogenic autoamplifying loop induced by EREG [32]. The expression of EREG may be considered a predictive biomarker of response to anti-EGFR therapies [68]. Here, we showed that the direct inhibition of EREG is indeed associated with resistance to CTX and/or irradiation, which recapitulates the phenotype of Cav1-expressing cells. Whether the suppression of EREG is dependent on EGFR sequestration at the cell membrane or the result of Cav1 repression requires further investigation. Nevertheless, EREG is a key target of Cav1-mediated cell resistance to CTX-chemotherapy.

Very recently, Cav1 was identified as a regulator of YAP activity [69]. Forty-two percent of HPV-negative HNSCC presents alterations in the HIPPO pathway [70]. Amplification of YAP and TAZ is found in 5% and 9% of HNSCC [70], respectively. EGFR activation leads to the nuclear translocation of YAP [71], resulting in the upregulation of AREG, EREG, TGF and EGFR [72]. This suggests an autocrine loop between both the EGFR and HIPPO pathways. Thus, YAP/TAZ might also be involved in the autoamplifying loop involving EREG. We have demonstrated here that Cav1-induced YAP activation requires the suppression of EREG. To our knowledge, this is the first study showing that EREG might repress YAP expression and activity. YAP activation confers to cells proliferative and motile advantages (through the activation of canonical target genes such as CTGF, CYR61, AXL, COL4A3, ITGB2, CCNE2, CDK2, BIRC5 and SOX9). It was also identified as a biomarker of resistance to CTX [37,70], cisplatin [73] or radiotherapy [74]. In accordance, the overexpression of YAP and its constitutively active mutant S127A renders cells resistant to CTX/irradiation treatment. Although the regulation of YAP by EREG deserves further study, our data clearly show that Cav1 promotes resistance to CTX/irradiation through the Cav1/EREG/YAP axis.

Finally, we have shown that high Cav1 expression in tumors is associated with locoregional relapse and worse prognosis. This study complements our prior paper, which showed that low or no Cav1 expression was observed in metastasis-prone HNSCC, and was also correlated to poor prognosis. The results published by Jung et al. [13] were obtained in a different cohort of patients. It was designed to identify molecular markers able to discriminate patients at high metastatic risk as the first recurrence event within 3 years after treatment, or patients with no progression at all during a 3-year follow-up period. Thus, low or no expression of Cav1 (characterizing metastatic patients) was indeed correlated with adverse prognosis when compared to the non-metastatic patient. In the present study, the correlation between Cav1 levels, relapse and overall survival has been determined in a representative cohort of patients, including both locoregional relapses and metastic events. Here, Cav1 overexpression is clearly associated only with locoregional relapses. Both studies are complementary and show that this independent prognostic biomarker could help to identify subgroups of advanced HNSCCs at higher risk of recurrence either locally or at distant sites. Both are a bad prognosis operating through different pathways, and should be taken into consideration for better risk stratification. Our tumor biobank includes only resected specimens and not small biopsy samples. As no prospective evidence supports single-agent CTX combined in the post-operative setting, we were not able to establish a group of patients treated with CTX-radiotherapy. Thus, we used human tissue samples derived from patients treated with adjuvant radiotherapy ± cisplatin. Although this might appear to be a limitation of our study, our purpose was to explore the prognostic value of the Cav1/EREG/YAP axis regardless of treatment. However, Cav1 detection might also be taken into consideration in the future in the clinic, not only to help clinicians choose more appropriate therapeutic strategies, but also to predict the success of the approach by taking into account probable radio-, CTX- or chemoresistance. As immunotherapies are new SOCs in the R/M setting, further studies will be needed to determine if Cav1 might also play a role in immune escape. Reduced basal growth/survival/motility following Cav1 expression seems to put cells into a state in which they can resist the treatment. Data tend to explain why patients displaying tumors with high levels of Cav1 relapse a few years after treatment. This also clearly indicates the inefficacy of EGFR-targeting drugs in Cav1-expressing tumors. Altogether, our observations suggest that a high expression of Cav1 may be predictive of a locoregional relapse of LA-HNSCC involving the Cav1/EREG/YAP axis.

## 6. Conclusions

Altogether, our work showed that Cav1 expression conferred surviving, growing and motile capacities that protect cells against the combination of CTX-radiotherapy. The protecting effects of Cav1 are mediated by the Cav1/EREG/YAP axis. High expression of Cav1 was predictive of locoregional relapse of LA-HNSCC. Cav1 should be taken into consideration in the future as a prognosis marker to identify the subgroup of advanced HNSCC at higher risk of recurrence, but also to help clinicians to choose the more appropriate therapeutic strategies.

## Data Availability

No new data were created or analyzed in this study. Data sharing is not applicable to this article.

## Supplementary Materials

The following are available online at https://www.mdpi.com/article/10.3390/cancers13123038/s1, Table S1. Antibodies, Table S2. Baseline patient characteristics, Data S1–S3. Cav1 expression in human HNSCC primary tumor tissues 1.
