# Supplementary material for: Cav1/EREG/YAP Axis in the Treatment Resistance of Cav1-Expressing Head and Neck Squamous Cell Carcinoma
Source: Cancers (Basel). 2021 Jun 18;13(12):3038. doi: 10.3390/cancers13123038 (PMC8235528; doi:10.3390/cancers13123038)

cancers

MDPI

Supplementary Materials: Cav1/EREG/YAP Axis in the Treatment Resistance of Cav1-Expressing Head and Neck Squamous Cell Carcinoma

Mickaël Burgy, Aude Jehl, Ombline Conrad, Sophie Foppolo, Véronique Bruban, Nelly Etienne-Selloum, Alain C Jung, Murielle Masson, Christine Macabre, Sonia Ledrappier, Hélène Burckel, Carole Mura, Georges Noël, Christian Borel, François Fasquelle, Mihaela-Alina Onea, Marie-Pierre Chenard, Alicia Thiéry, Monique Dontenwill and Sophie Martin

Table S1. Antibodies.

Antibodies	Species	Dilution	Suppliers
Caveolin-1 (N-20, sc-894)	Rabbit	1:1000	Santa Cruz Biotechnology
GAPDH (MAB374)	Mouse	1:10000	EMD Millipore©
Cleaved PARP (#9541, Asp214)	Rabbit	1:1000	
TAZ (D3I6D)	Rabbit	1:1000	
YAP (14074S)	Rabbit	1:1000	
pYAP S127 (D9W2I)	Rabbit	1:1000	
EGFR (D1D4J) XP®	Rabbit	1:1000	Cell Signaling Technology©
p44/42 MAPK (#9102)	Rabbit	1:1000	
Phospho-p44/42 MAPK (#9101, Thr202/Tyr204)	Rabbit	1:1000	
AKT (#9272)	Rabbit	1:1000	
Phospho-AKT (#4060, D9E, Ser473)	Rabbit	1:1000	
Anti-mouse IgG HRP-conjugate<br>Anti-rabbit IgG HRP-conjugate	Goat	1:10000	Promega©

Supplemantary data 1

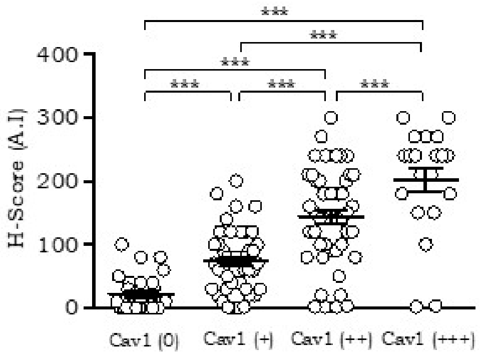

Supplemantary data 2

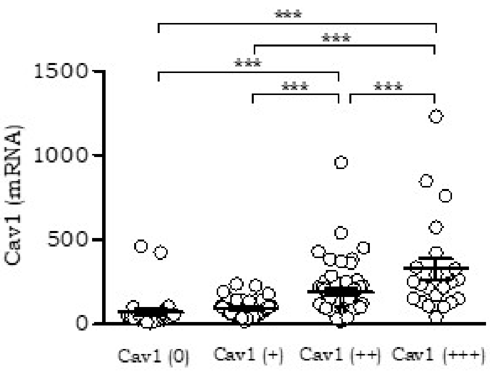

Supplemantary data 3

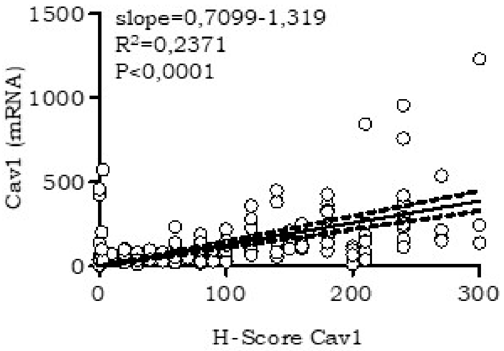

Data S1–S3: Cav1 expression in human HNSCC primary tumor tissues 1, The H-score was calculated as a percentage of cells stained for Cav1 (0, 1, 2 or 3) using the formula (1 × (% cells 1) + 2 × (% cells 2) + 3 × (% cells 3)). Histograms show the Cav1 H-score in each subgroup identified by IHC. **2,** Histograms show the mRNA expression level of Cav1 in each subgroup identified by IHC. **3,** Correlation curve between the mRNA expression level of Cav1 and the Cav1 H-score. Each bar represents the mean±SEM with ****p* < 0.001.

Original western blots:

Original blot (overlay PM marker+image) - FIGURE 1B

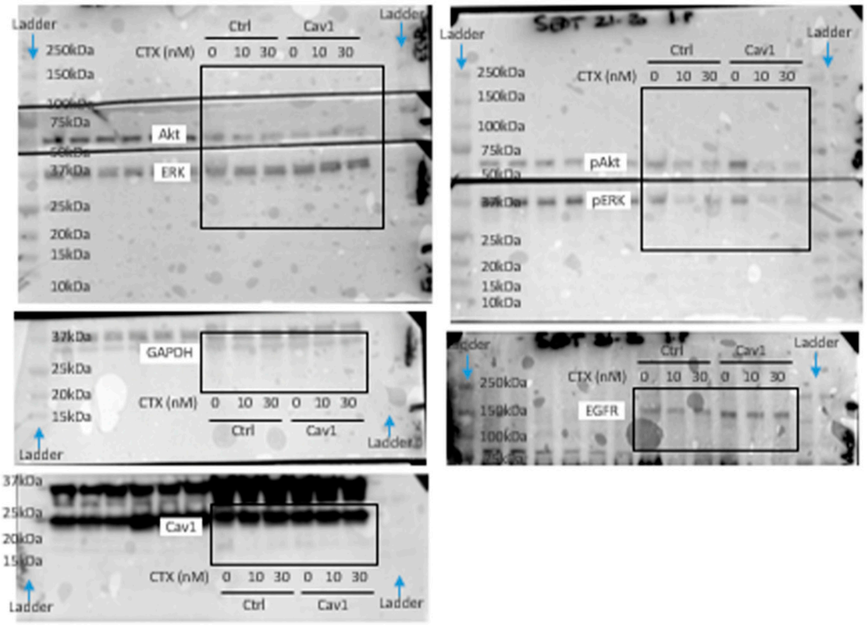

Original blot (overlay PM marker+image) - FIGURE 2E

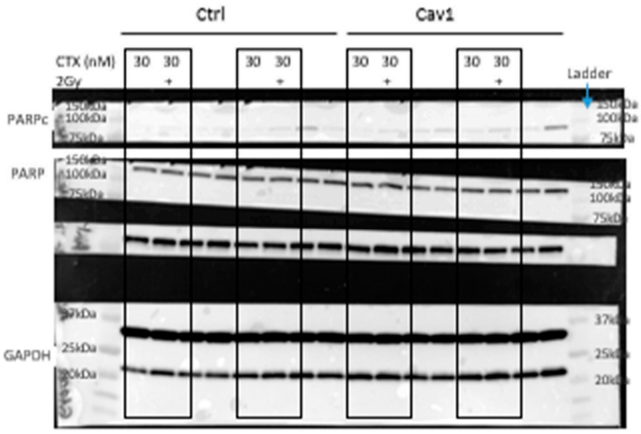

Original blot (overlay PM marker+image) - FIGURE 4B (GAPDH)

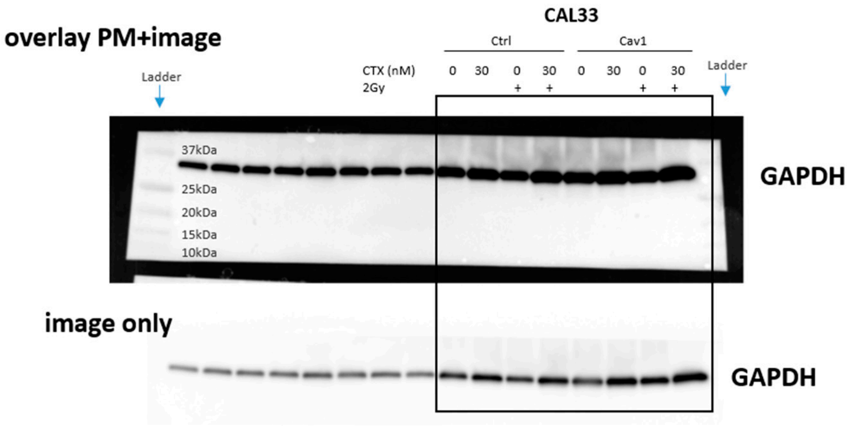

Original blot (overlay PM marker+image) - FIGURE 5A

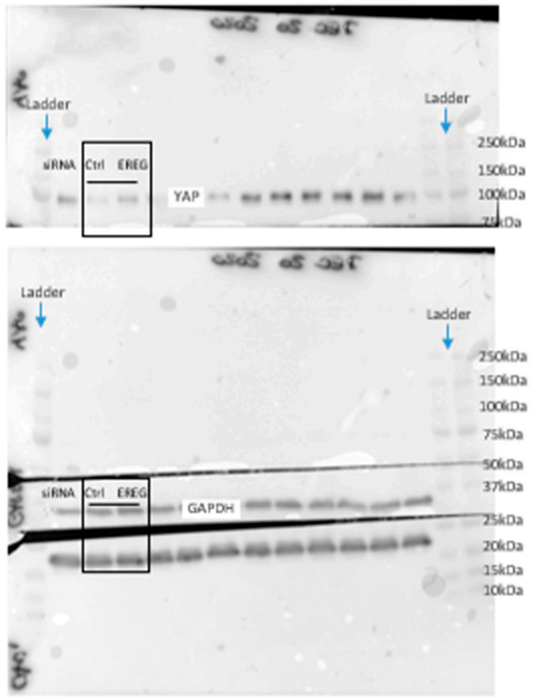

Original blot (overlay PM marker+image) - FIGURE 5B

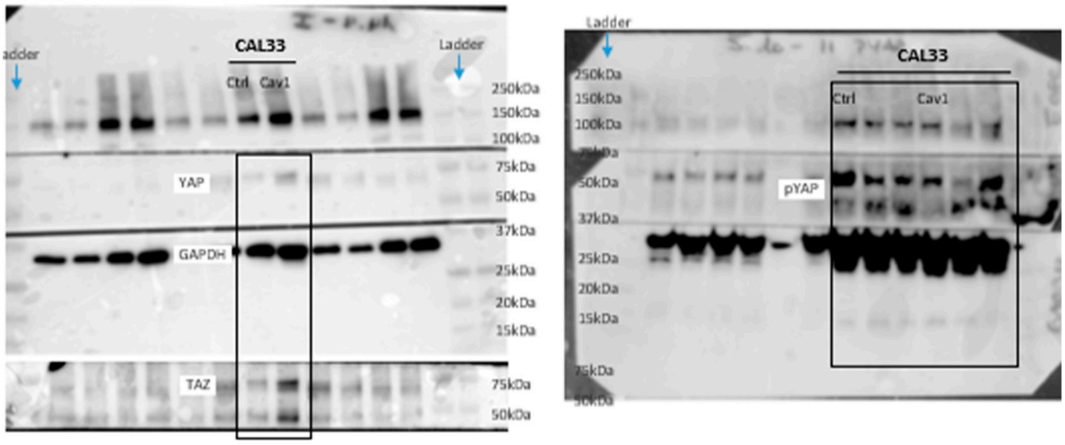

Original blot (overlay PM marker+image) - FIGURE 5D

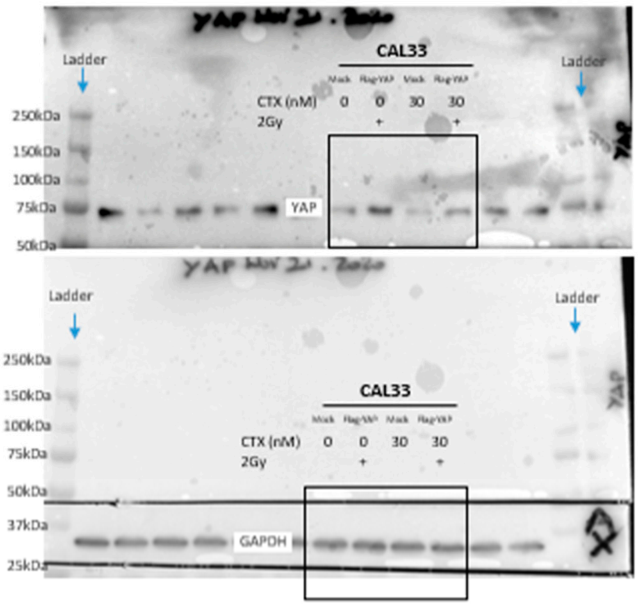

Supplement: Supplementary file 1 [file cancers-13-03038-s001.zip › cancers-1227836-supplementary conversion.pdf]
